# Vitamin D status in cats with cardiomyopathy

**DOI:** 10.1111/jvim.15833

**Published:** 2020-06-17

**Authors:** Wendy A. Ware, Lisa M. Freeman, John E. Rush, Jessica L. Ward, Andrew J. Makowski, Min Zhang

**Affiliations:** ^1^ Department of Veterinary Clinical Sciences College of Veterinary Medicine, Iowa State University Ames Iowa USA; ^2^ Department of Clinical Sciences Cummings School of Veterinary Medicine, Tufts University North Grafton Massachusetts USA; ^3^ Heartland Assays LLC & Metabolic Technologies, Inc. Ames Iowa USA; ^4^ Department of Statistics, College of Liberal Arts and Sciences Iowa State University Ames Iowa USA

**Keywords:** 25(OH)D, 3‐epimer, feline, myocardial disease

## Abstract

**Background:**

Low vitamin D concentrations have been associated with advanced heart disease and poorer outcomes in people and dogs. Vitamin D status typically is assessed by serum 25(OH)D concentration. However, cats also produce notable amounts of a C‐3 epimer of 25(OH)D (3‐epi).

**Hypothesis/Objectives:**

Determine if vitamin D status, estimated by 25(OH)D_3_ alone or combined with 3‐epi (summation vitD), is lower in cats with cardiomyopathy (CM) compared to clinically normal (N) cats and if indicators of disease severity are associated with vitamin D status.

**Animals:**

Privately owned cats, 44 with CM and 56 N.

**Methods:**

Cross‐sectional observational study using clinical and echocardiographic findings, diet history, and serum 25(OH)D_3_ and 3‐epi measurements.

**Results:**

Cat age was negatively related to vitamin D status. Summation vitD was lower in CM cats (median = 47.1 ng/mL) compared to N cats (median = 58.65 ng/mL) both before (*P* = .03) and after (*P* = .04) accounting for age. However, 25(OH)D_3_ became nonsignificant between CM and N cats after age was included. Summation vitD was related positively to survival time and fractional shortening (FS), but negatively to left atrial enlargement (LAE) severity, both before and after accounting for age. For 25(OH)D_3_ alone, only survival time and FS remained significant after including age.

**Conclusions and Clinical Importance:**

We report 25(OH)D_3_ and 3‐epi concentrations in CM and N cats. Age had an important (negative) relationship to vitamin D status. After accounting for age, summation vitD was lower in CM cats. Vitamin D status was related positively to survival time and FS, but negatively to LAE severity.

**Abbreviations**
1, 25[OH]_2_D_3_: calcitriol
25(OH)D: calcidiol, 25‐hydroxyvitamin D_3_ (± D_2_)
3‐epi: C‐3 epimer of 25(OH)D, 3‐epi‐25(OH)D_3_
CHF, congestive heart failure; CM: cardiomyopathy
CV: cardiovascular
DMVD: degenerative (myxomatous) mitral valve disease
Echo: echocardiography
FS: fractional shortening
HCM: hypertrophic cardiomyopathy
HOCM: hypertrophic obstructive cardiomyopathy
ISU: Iowa State University
LAE: left atrial enlargement
LC/MS/MS: liquid chromatography‐tandem mass spectrometry
LV: left ventricular
N: clinically normal
RCM: restrictive cardiomyopathy
Summation vitD: sum of 25(OH)D_3_ and 3‐epi concentrations
UHPLC: ultra‐high pressure liquid chromatography
vitamin D_3_: cholecalciferol

## 1 INTRODUCTION

Decreased concentrations of vitamin D (as assessed by serum 25(OH)D concentration) have been observed in people with systemic hypertension and other cardiovascular (CV) conditions that often lead to congestive heart failure (CHF). Low vitamin D status has been associated with disease progression and poorer prognosis in people with CV disease and CHF, including those with diastolic dysfunction and preserved ejection fraction.^1^, ^2^, ^3^ Vitamin D is thought to have a protective effect on the CV system through regulatory influences on blood pressure, as well as on cardiac, endothelial, and smooth muscle cell function.^1^ Many studies report 25(OH)D measurement without specifying whether 25(OH)D_3_ only has been measured, or both 25(OH)D_3_ and 25(OH)D_2_ (ie, total 25(OH)D or 25(OH)D_2/3_). As a point of clarification, we use “25(OH)D” in reference to reports using the same general term; otherwise, we employ the more specific terminology.

Two small studies in dogs with degenerative (myxomatous) mitral valve disease (DMVD) or dilated cardiomyopathy (CM) showed lower serum 25(OH)D concentrations in those with heart failure (or advanced preclinical disease) compared to normal dogs.^4^, ^5^ Associations between low vitamin D status and more advanced cardiac remodeling, worse function, and negative clinical outcome were noted. It is not known whether vitamin D status is lower in cats with CM compared to normal cats, and if so, whether an association exists between vitamin D status and disease severity or outcome.

Although the most biologically active form of vitamin D is calcitriol (1, 25(OH)_2_D_3_), its immediate precursor, 25(OH)D (or calcidiol), is more stable in the circulation. Therefore, serum 25(OH)D concentration typically has been used to assess vitamin D status, especially in people and dogs. Dietary precursors of 25(OH)D_2/3_ are cholecalciferol (vitamin D_3_) from animal food sources, and ergocalciferol (vitamin D_2_) from plant sources; in cats and dogs, contributions from vitamin D_2_ are minimal. These species rely on dietary intake because they cannot synthesize vitamin D_3_ within the skin after ultraviolet light exposure.^6^

An additional consideration regarding vitamin D status in cats is the formation of a C‐3 epimer of 25(OH)D_3_ (3‐epi‐25(OH)D_3_ or 3‐epi). This metabolite is known to occur in people, cats, rats and, recently, dogs.^6^, ^7^, ^8^, ^9^ Circulating concentrations of 3‐epi, compared to 25(OH)D_3_, appear to be much higher in cats than in people and dogs.^8^, ^9^ Epimeric forms of vitamin D are thought to have at least some biological activity, although how much is unclear.

We hypothesized that vitamin D status would be lower in cats with CM compared to clinically normal (N) cats. The main purpose of our study was to determine if vitamin D status, as assessed by serum 25(OH)D_2/3_ or the summation of 3‐epi and 25(OH)D_3_ concentrations (summation vitD), is lower in cats with spontaneously occurring CM compared to clinically healthy cats. We also sought to identify whether certain clinical variables or indicators of disease severity are associated with vitamin D status.

## 2 MATERIAL AND METHODS

Our study was a cross‐sectional observational study. The CM group was recruited from privately owned cats presented for veterinary care at the Veterinary Medical Centers of Iowa State University (ISU) and Tufts University between November 2016 and April 2018. Cats were entered into the CM group based on echocardiographic evidence for hypertrophic cardiomyopathy (HCM) or hypertrophic obstructive cardiomyopathy (HOCM), restrictive cardiomyopathy (RCM), or other forms of CM, and left atrial enlargement (LAE), as an indication of at least moderate disease severity. Cats were excluded if they had clinical, client‐reported, or prior medical record evidence for serious systemic disease at enrollment, including severe hypertension (>180 mm Hg, systolic), hyperthyroidism, severe chronic kidney disease, active inflammatory bowel disease, systemic neoplasia, or widespread infectious or inflammatory disease. The N group was recruited at ISU during approximately the same time period and was composed of cats owned by students, staff, and local community residents. As an entry criterion, all cats were being fed commercial cat food (not a homemade diet) as the primary diet. Owners completed detailed diet and health history (see Supporting Information) and informed consent forms. The study was approved by the animal care and use committees at both universities.

Clinical data collected included signalment, body weight (kg), body condition (1‐9), and muscle condition (1‐4; https://www.wsava.org/Guidelines/Global-Nutrition-Guidelines) scores, CV examination findings, indirect systolic blood pressure measurement (mm Hg, Doppler method, average of at least 3‐5 readings) and echocardiography (echo; 2‐D, M‐mode, color flow, and spectral Doppler). Echo studies were performed by a board‐certified cardiologist or a cardiology resident under the direct supervision of a board‐certified cardiologist. Cats with CM were classified according to the American College of Veterinary Internal Medicine (ACVIM) heart disease staging system proposed for cats, which is similar to that used for dogs with DMVD.^10^ Stage B2 cats had evidence for CM and atrial enlargement, but had not developed CHF. Stage C cats had signs of CHF either at presentation (stage C‐active CHF) or in the past, which resolved with heart failure treatment (stage C‐compensated). A venous blood sample (1‐2 mL) also was obtained for 25(OH)D_2/3_ and 3‐epi measurement; serum was separated and then immediately frozen and stored at −80°C until analyzed. Screening serum biochemistry and thyroid tests were not performed in all cats at enrollment because of financial constraints. For most cats, however, where that information was not otherwise available, we measured creatinine, blood urea nitrogen, electrolyte, and T_4_ concentrations. Follow‐up to determine survival status at study closure (February 1, 2019) was by phone or email contact with cat owners or referring veterinarians.

Assays for 25(OH)D_3_, 25(OH)D_2_, and 3‐epi were performed by a commercial laboratory (Heartland Assays, Ames, Iowa) that is Vitamin D External Quality Assessment Scheme (DEQAS) certified using liquid chromatography‐tandem mass spectrometry (LC/MS/MS; Agilent 6460 MS/MS with ESI source, assayed in positive mode; Aglient Technologies, Santa Clara, California) according to previously described methods.^11^, ^12^, ^13^ Briefly, serum samples along with standard curve and controls were protein precipitated using 0.2 M zinc sulfate solution, vortexed, followed by methanol addition and d3‐25(OH)D_2_/d3‐25(OH)D_3_ internal standards. All samples and controls were vortexed followed by hexane addition then capped, vortexed, and centrifuged. Organic layer was transferred and dried. All standards, controls, and samples then were reconstituted with LC/MS grade methanol and water (both containing 0.1% formic acid), and loaded onto the autosampler for analysis. Analyte separation was achieved using ultra‐high pressure liquid chromatography (UHPLC) with a pentyl fluoro phenyl (PFP) column (Agilent 1290 infinity UHPLC and Poroshell Pentyl fluoro phenyl [PFP 2.1 × 100 mm 2.7 μm] column; Aglient Technologies) to selectively separate 3‐epi‐25(OH)D_3_ from the 25(OH)D_3_. Assay accuracy was determined to be >95%, based on National Institute of Standards and Technology‐certified standard assessment for 25(OH)D_2_, 25(OH)D_3_, and 3‐epi‐25(OH)D_3_.^12^, ^13^

Diet histories provided by cat owners, along with more specific details obtained as necessary by follow‐up owner contact, were used to estimate vitamin D intake from commercial cat foods eaten by individual cats. Intake from treats was minimal for all cats, and thus vitamin D intake from treats or table food was not included. Manufacturers of specific commercial diets being fed were contacted for data on vitamin D content in those diets. Estimated daily vitamin D intake (IU cholecalciferol/kg body weight) was calculated from the estimated daily amount of each specific diet eaten by individual cats, in conjunction with the manufacturer's reported vitamin D content for the diet.

Clinical variables included for analysis were: age, sex, body weight, body condition score, muscle condition score, diagnosis category (N, HCM, HOCM, or other CM), heart disease category (N = none, or ACVIM stage B2, stage C‐compensated, or stage C‐active CHF), number of days since onset of CHF (or 0, for N and stage B2 CM), presence or absence of pleural effusion at enrollment, presence or absence of an arrhythmia, survival time (days from enrollment to death or study end date, whichever occurred first), presence or absence of an arterial thromboembolic event (historical or during study period), and estimated daily vitamin D intake (IU/kg). Echocardiographic findings analyzed were: LAE severity category (right parasternal long‐axis view, systole: none, LA diameter < 17 mm; mild, 17‐18 mm; moderate, 19‐22 mm; or severe, >22 mm), sum of diastolic interventricular septal and left ventricular (LV) free wall thicknesses (as estimate of LV hypertrophy), LV fractional shortening (FS), and end‐diastolic LV internal diameter. The latter 3 variables were obtained from the right parasternal short‐axis view. To help further define the influence of age on relationships between vitamin D status and certain clinical variables, we also categorized cats into 3 age groups (1: ≤5 years old, n = 26; 2:6‐9 years old, n = 22; and 3: ≥10 years old, n = 52).

Statistical tests were performed using R, an open‐source software (R Core Team [2018], R Foundation for Statistical Computing, Vienna, Austria, https://www.R-project.org/). One‐way analysis of variance (for continuous factors) and Fisher's exact test (for categorical factors) were used to compare the CM and N populations. Pearson correlation tested for relationship between serum concentrations of 25(OH)D_3_ and 3‐epi. Multiple linear regression modeling to include all potential explanatory variables was explored but multicollinearity among variables confounded this approach. Therefore, backward selection was performed to eliminate the full model. In addition, linear regressions were conducted separately on each of the clinical variables to test for their relationships with 25(OH)D_3_, 3‐epi alone, or summation vitD concentrations individually. To account for the effect of age, this important factor was included together with each clinical variable in further regression analysis. Model diagnostics were conducted to confirm assumptions of linear regression. The Wilcoxon Rank Sum test was used to compare estimated dietary vitamin D intake in CM and N cats, because of skewness of data distribution. Possible relationships between vitamin D intake and 25(OH)D_3_, 3‐epi, or summation vitD were assessed by Pearson correlation. *P* values <.05 were considered significant.

## 3 RESULTS

Forty‐four CM cats were enrolled (29 at ISU and 15 at Tufts). Thirty‐eight of 44 CM cats (86%) had a history of CHF. The majority (68%) of these had active signs of congestion at presentation, although some were well compensated on heart failure treatment. We enrolled 56 N cats during the same time period, 44 of which were sex‐ and age‐matched (±6 months) to the CM cats. Analyses reported here included the 44 CM cats and 56 N cats. Comparison of study group characteristics identified no significant differences between CM and N groups, except for diagnosis category (Table 1).

**TABLE 1:** Study population characteristics of cats with cardiomyopathy and clinically normal controls

	Cardiomyopathy cats (n = 44)	Clinically normal cats (n = 56)
Age (y), median (range)	10.5 (0.6‐17.5)	9.25 (1‐23)
Sex		
Male	36 neutered, 1 intact	37 neutered
Female	7 spayed	17 spayed, 2 intact
Body weight (kg), median (range)	4.9 (2.9‐8.6)	5.1 (2.3‐7.7)
Body condition score	5 (3‐8)	6 (2‐9)
Muscle condition score	4 (1‐4)	4 (1‐4)
Breeds		
Domestic shorthair/longhair	37	50
Other (purebred)	1 each: Maine Coon, Norwegian Forest, Persian, Ragdoll, Savanah, Siamese, and Sphynx cats	2 Bengals and 1 each: Himalayan, Persian, Siamese, and Turkish Van cats
Diagnosis category*		
N	0	56
HCM	20	0
HOCM	9	0
Other (includes 6 RCM, 6 “unspecified” CM, and 3 DCM)	15	0

Abbreviations: CM, cardiomyopathy; DCM, dilated cardiomyopathy; HCM, hypertrophic cardiomyopathy; HOCM, hypertrophic obstructive cardiomyopathy; N, normal; RCM, restrictive cardiomyopathy.

* *P* < .001. Other comparisons between CM and N groups showed no significant differences.

**FIGURE 1:**
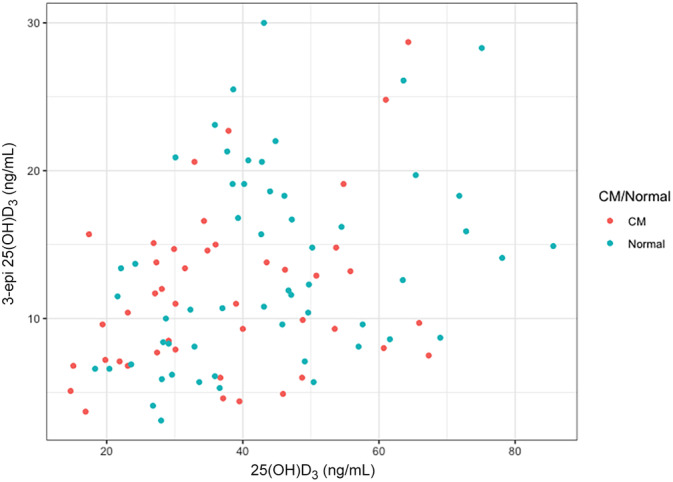
Scatterplot of serum 25(OH)D_3_ and 3‐epi‐25(OH)D_3_ concentrations, color‐grouped by CM status. Serum 25(OH)D_3_ and 3‐epi‐25(OH)D_3_ concentrations were positively correlated with each other (results for all CM and normal cats together shown); correlation coefficient = 0.35; *P* < .001. CM, cardiomyopathy

Serum concentrations of 25(OH)D_3_ and 3‐epi correlated positively with each other for all cats combined (Figure 1; correlation coefficient, 0.35; *P* < .001). Results were similar when CM (correlation coefficient, 0.32; *P* = .04) and N (correlation coefficient, 0.33; *P* = .01) cat groups were analyzed separately. We also evaluated the ratio of 3‐epi to summation vitD. No difference in mean ratio was found between CM cats and N cats. Table 2 contains summary statistics for these vitamin D measures. The concentration of 25(OH)D_2_ was below detection limits (<1.5 ng/mL) in all cats, and thus no analysis was performed.

**TABLE 2:** Summary statistics for selected continuous variables

Variable	Number of observations	Mean	Min.	25th percentile	Median	75th percentile	Max.
25(OH)D_3_ (ng/ml) (All cats)	100	41.04	14.7	29	38.8	49.83	85.6
CM cats	44	37.45	14.7	27.25	35.4	48.73	67.3
N cats	56	43.86	18.3	31.75	42.75	50.25	85.6
3‐epi (ng/mL) (All cats)	100	12.64	3.1	7.85	11.55	15.98	30
CM cats	44	11.57	3.7	7.43	10.7	14.63	28.7
N cats	56	13.48	3.1	8.25	12.1	18.38	30
Summation vitD (ng/mL) (All cats)	100	53.68	19.8	38.78	54.3	64.18	103.4
CM cats	44	49.02	19.8	37.9	47.1	59.78	93
N cats	56	57.34	24.9	40.58	58.65	65.53	103.4
3‐epi as % of (summation vitD) (All cats)	100	23.88	9.6	17.33	22.82	30	47.4
CM cats	44	24.28	9.6	18.64	24.28	30.33	47.4
N cats	56	23.57	10	17.13	22.7	29.77	41
Survival^a^ (d) (All cats)	90	369.52	4	131.25	346.5	616	831
CM cats	42	252.95	4	50.5	197	376.5	831
N cats	48	471.52	54	339	459	669	823
LV fractional shortening (%) (All cats)	99	47.68	11.8	42.45	49.6	55.75	70
CM cats	43	41.23	11.8	26.85	44.2	53.3	70
N cats	56	52.64	37.9	46.78	52.45	57.93	70
Estimated vitamin D intake (IU/kg) (All cats)	74	25.11	4.51	12.34	18.73	29.71	96.9
CM cats	33	32.08	5.45	18.18	22.5	35.85	96.9
N cats	41	19.5	4.51	10.5	15.5	24.53	61.7

Abbreviations: 3‐epi, 3‐epi‐25(OH)D_3_; CM, cardiomyopathy; LV, left ventricular; N, normal; Summation vitD, sum of 25(OH)D_3_ and 3‐epi concentrations.

^a^ Survival days were taken as right‐censored at study end date to include as many data points as possible.

To determine multiple linear regression models for 25(OH)D_3_ and summation vitD, we started with full models including all clinical variables. Because of the multicollinearity issue, and based upon the parsimony principle, we decreased the full models with backward elimination. For 25(OH)D_3_, only age, survival time, and FS remained; the latter 2 predictors were positively correlated (correlation coefficient 0.411). Therefore, from a practical standpoint, survival time was removed from the model, because this information could be more difficult to obtain than echo assessment of FS. The resulting model was: 25(OH)D_3_ = 35.77 − 0.98 × age + 0.31 × FS, with *R* ^2^ = 0.15. Hence, on average and with other conditions fixed, a 1 year increase in age is associated with 0.98 ng/mL decrease in 25(OH)D_3_ (*P* = .002) and a 1% increase in FS is associated with 0.31 ng/mL increase in 25(OH)D_3_ (*P* = .009).

Similarly, for summation vitD, backward selection decreased the model to factors age, muscle score, survival time, and FS; the latter 3 have *P* values between .05 and .1. After removing survival time for the same practical reason and backward selecting the model, we attained summation vitD = 47.86 − 1.2 × age + 0.37 × FS, with *R* ^2^ = 0.16. Thus, on average, each 1 year increase in age is associated with 1.2 ng/mL decrease in summation vitD (*P* = .001), whereas a 1% increase in FS is associated with 0.37 ng/mL increase in summation vitD (*P* = .009).

**FIGURE 2:**
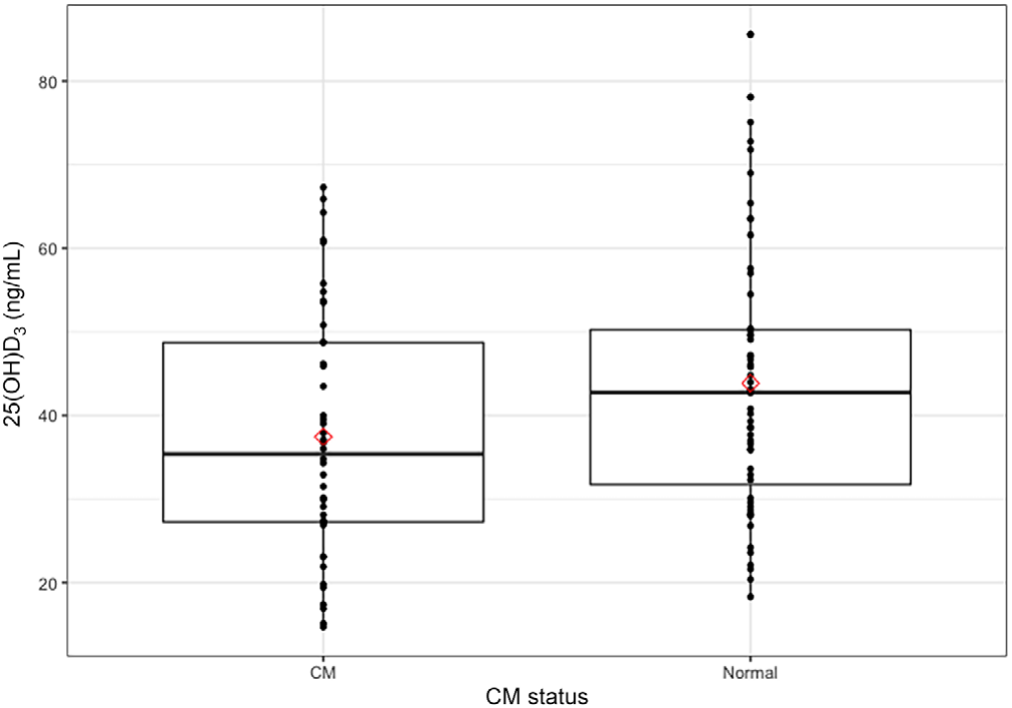
Box plots with data points of serum 25(OH)D_3_ concentrations for CM and N cats. Box plots show comparison between serum 25(OH)D_3_ concentrations for cats with CM and normal cats. After accounting for age, the difference became nonsignificant (*P* = .07). Bar inside each box indicates median, with lower and upper edges marking 25th and 75th percentiles, respectively. Mean values indicated by red diamonds. CM, cardiomyopathy

**FIGURE 3:**
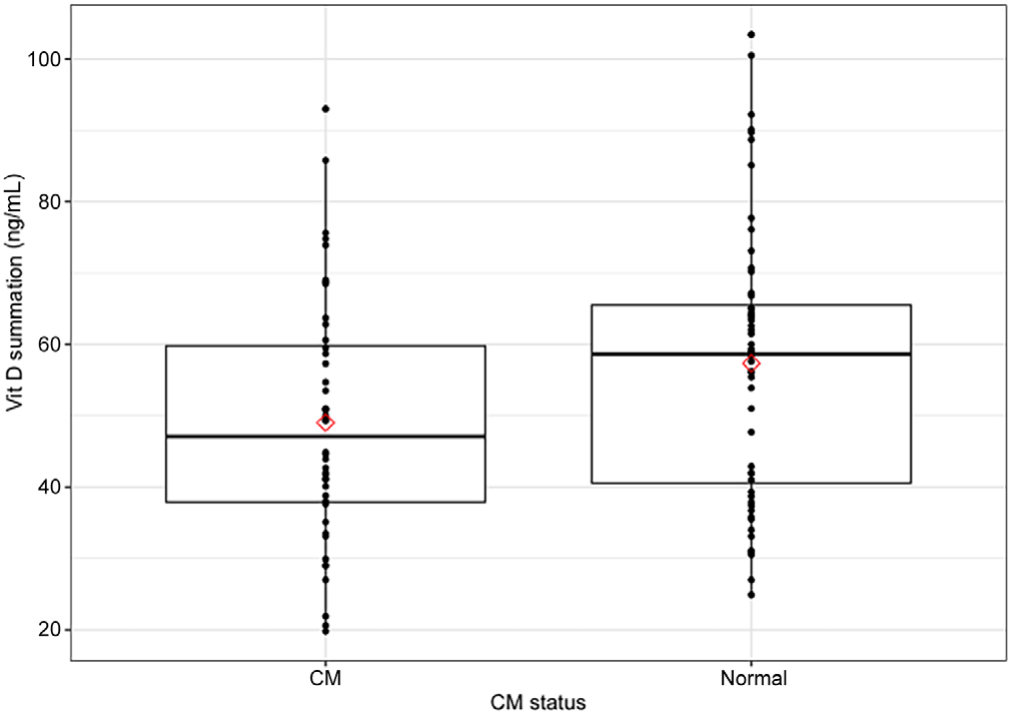
Box plots with data points of summation vitamin D concentrations for CM and N cats. Box plots show comparison between summation vitamin D (serum 25(OH)D_3_ plus 3‐epi‐25(OH)D_3_ concentrations) for cats with CM and normal cats. Difference is significant, both before (*P* = .03) and after (*P* = .04) accounting for age. Bar inside each box indicates median, with lower and upper edges marking 25th and 75th percentiles, respectively. Mean values indicated by red diamonds. CM, cardiomyopathy

In addition to modeling vitamin D measurements with clinical predictors combined, their associations with each individual predictor, CM status (CM versus N), age, sex, and the other clinical variables, were assessed. Significant results are listed in Table 3, including model coefficients (*β*), *P* values, and *R* ^2^ values. Age (as a continuous variable) was negatively related to 25(OH)D_3_. After accounting for age, the difference in 25(OH)D_3_ between CM and N groups became nonsignificant (Figure 2). No relationship was found between sex and 25(OH)D_3_. For 3‐epi, CM status, age, and sex were nonsignificant. For summation vitD, as with 25(OH)D_3_, age was a significant factor (*P* = .001). In contrast to 25(OH)D_3_ alone, summation vitD was significantly different between CM and N cats even after including age in the model (*P* = .04; Figure 3).

**TABLE 3:** Significant results of regression analyses between vitamin D measures and clinical variables

Variable	Model results	25(OH)D			Summation vitD		
		Alone	After accounting for age		Alone	After accounting for age	
			Variable	Age		Variable	Age
CM versus N (N: baseline)	*β*	−6.406	−5.624	−0.947	−8.321	−7.359	−1.164
	*P*	.04	.06	.003	.03	.04	.002
	*R*^2^	0.042	0.128		0.049	0.140	
Age	*β*	−0.996	—	—	−1.228	—	—
	*P*	.002			.001		
	*R*^2^	0.096	—		0.102	—	
Survival time	*β*	0.022	0.017	−0.844	0.027	0.019	−1.096
	*P*	.001	.02	.01	.001	.02	.009
	*R*^2^	0.117	0.175		0.115	0.183	
Left atrial enlargement	*β*	−2.844	−2.442	−0.916	−3.921	−3.431	−1.116
	*P*	.04	.07	.004	.02	.03	.003
	*R*^2^	0.043	0.123		0.058	0.140	
Fractional shortening	*β*	0.307	0.308	−0.977	0.364	0.365	−1.202
	*P*	.01	.009	.002	.01	.009	.001
	*R*^2^	0.063	0.155		0.062	0.159	
Diagnosis category: other CM^a^ versus N (N: baseline)	*β*	−8.067	−6.493	−0.938	−10.914	−8.990	−1.146
	*P*	.08	.14	.004	.05	.09	.003
	*R*^2^	0.048	0.128		0.058	0.142	
Heart failure severity^b^	*β*	−2.221	−1.855	−0.945	−3.127	−2.679	−1.155
	*P*	.07	.11	.003	.03	.05	.002
	*R*^2^	0.034	0.119		0.047	0.136	

Abbreviations: CM, cardiomyopathy; N, normal; Summation vitD, sum of 25(OH)D_3_ and 3‐epi concentrations.

^a^ See Table 1 and text for further details.

^b^ See text for further details.

Positive associations were seen between summation vitD and the variables survival days and FS, and negative association was seen with LAE severity, both before and after accounting for age. The diagnosis category of other CM showed a significantly smaller mean summation vitD concentration compared to N cats before accounting for age but, with age in the model, the relationship was nonsignificant. Further analysis using the filter of previously defined age groups indicated that older cats (age group 3) with other CM had lower summation vitD, compared to N cats (*P* = .04). Heart failure severity had a negative relationship with summation vitD before age was included in the model (*P* = .03); but, this difference became only borderline after accounting for age (*P* = .05). When the age group filter was applied, a negative relationship between heart failure severity and summation vitD was identified in the young cat group (1). Only 8/24 cats in this age group had decompensated CHF, but summation vitD was extremely low (20.6 ng/mL) in 1 of them. When this outlier was excluded from analysis, heart failure was no longer a significant variable for group 1 cats. For 25(OH)D_3_ by itself, significant positive relationships were found with survival days and FS, and a negative relationship was found with LAE severity. However, after accounting for the effect of age, only survival time and FS remained significant. Diastolic LV internal diameter was positively associated with age, but not with 25(OH)D_3_ or summation vitD after age was included in the model.

We intended to exclude cats with more than minor azotemia from our study but we discovered after enrollment that a small number of geriatric cats with emergency presentations for CHF actually had more marked azotemia. Therefore, we included azotemia status in the analysis using the categorization criteria: none, serum creatinine concentration (creat) < 2.1 mg/dL or blood urea nitrogen concentration (BUN) < 29 mg/dL; mild, creat 2.2 to 2.9 mg/dL or BUN 30 to 40 mg/dL; moderate, creat 3 to 4 mg/dL or BUN 41 to 50 mg/dL; and severe, creat >4 mg/dL or BUN >51 mg/dL. Azotemia status was positively associated with age, but not with 25(OH)D_3_ or summation vitD after age was included in the model.

Daily vitamin D intake was estimated for 74/100 cats. For the remaining cats, insufficient information about specific food and flavor, daily amount consumed, or the food's vitamin D content precluded making reasonable estimates of vitamin D intake. The CM group did have higher estimated vitamin D intake compared to the N group (median = 22.6 IU/kg and 15.5 IU/kg, respectively; *P* = .002). However, no correlation was found between vitamin D intake and serum 25(OH)D_3_, 3‐epi, or summation vitD for all cats in which data were available. Neither was correlation found when CM and N cats were tested separately.

## 4 DISCUSSION

A striking finding in our study was the important effect of cat age on vitamin D status, both for 25(OH)D_3_ alone and summation vitD, with older cats more likely to have lower vitamin D status. To our knowledge, this age influence on 25(OH)D and summation vitD has not been reported previously in cats. In dogs with DMVD, although an age difference was noted between control group and stage C or D heart failure group, no correlation was found between 25(OH)D concentration and age.^5^ The other published study involving dogs with clinical cardiac disease also found no significant association between age and 25(OH)D.^4^ Although the CHF group was older than the controls in that study, univariate linear regression of all dogs across both populations showed no significant relationship between age and 25(OH)D concentration. Those investigators also found that multivariable regression analysis controlling for age indicated that the significant difference in 25(OH)D concentration between groups was maintained.^4^ Likewise, in another study of 69 dogs with cancer compared to 23 healthy dogs, 25(OH)D concentration was lower in the different cancer groups, but no age effect was identified on 25(OH)D concentration.^14^ Studies in people have had conflicting findings. Given comparable intake of vitamin D, some report no association between age and 25(OH)D concentration.^15^, ^16^ However, increased vitamin D intake and sun exposure do lead to higher 25(OH)D concentrations. In contrast, a study involving adults >60 years of age that focused on the relationship of vitamin D status to antioxidant status did find that median age was older in people with baseline 25(OH)D concentration < 50 nM/L (ie, <20 ng/mL) compared to those with higher 25(OH)D concentrations, although there was much overlap.^17^ Follow‐up measurement 8 years later within the same population identified a significant decrease in median 25(OH)D, despite comparable dietary vitamin D intake and percentage of individuals taking vitamin D supplements.

In our study, the 3‐epi metabolite was present in substantial amounts in both CM and N cats, thus it appears to be an important metabolite in this species. The fact that 3‐epi alone, as well as the ratio of 3‐epi to summation vitD (or 3‐epi as percentage of summation vitD), was not different between CM and N cats suggests that formation of this metabolite is not affected by CM status. In our cat population, 3‐epi comprised a mean of almost 24% of summation vitD concentration. In another study, a mean 3‐epi concentration of 20.9 ng/mL (LC‐MS method) was reported in a small group of adult male cats, and 3‐epi represented a mean of about 56% of 25(OH)D_3_ concentration.^8^ We found a smaller, but still substantial, concentration of 3‐epi in our larger and more diverse cat population. Expressed similarly, our population's 3‐epi concentration represented a mean of 33.2% of 25(OH)D_3_ concentration alone. The prior study also found that increased dietary vitamin D intake led to an increase in serum 3‐epi concentration, but no significant change in 25(OH)D_3_.^8^ Because our study measured vitamin D metabolites at only 1 point in time, we have no basis for comparison.

The concentration of the 3‐epi metabolite in cats is much higher and more consistently identified than that reported in people and dogs. Although, 3‐epi was identified in 87.2% of dogs sampled, concentrations were below the quantification limit in 40% of these and median 3‐epi concentration was only 5.2 nM/L (2.08 ng/mL).^9^ In people, 3‐epi was identifiable in variable proportions (0%‐100%) of populations studied and racial differences were found.^18^ In 1 study, only 33% of Caucasians and 15% of African Americans had quantifiable (>1.4 ng/mL) 3‐epi concentrations and these were low.^7^ Mean 3‐epi concentrations, when quantifiable, were 2.12 ng/mL and 2.16 ng/mL in Caucasians and African Americans, respectively. Furthermore, 3‐epi constituted only 3.23% and 2.25%, respectively, of the total of 25(OH)D_3_ and 3‐epi concentrations (ie, summation vitD). An earlier review of humans calculated a weighted mean across studies in adults, estimating a median 3‐epi of 1.72 ng/mL, with 3‐epi constituting approximately 6.1% of summation vitD.^18^ As in our study of cats, 3‐epi concentrations in people and dogs have correlated positively with 25(OH)D_3_ concentrations.^7^, ^9^

Given the concentrations of 3‐epi in cats, we think it reasonable to consider the sum of 3‐epi and 25(OH)D_3_ concentrations as a potential clinical index of vitamin D status in this species. Such an approach also has been used in people^7^ and, in view of the higher circulating 3‐epi concentration in cats compared to people, this approach seems especially relevant. Studies in rodents and in vitro studies suggest that 3‐epi and the epimeric form of calcitriol bind to vitamin D binding protein at 36% to 46% of the affinity of the parent molecules, and to vitamin D receptors at 2% to 3% of 25(OH)D and calcitriol, respectively.^7^ The epimeric form of calcitriol was shown to suppress parathyroid hormone secretion similarly to the nonepimeric form, despite its lesser capacity to bind the vitamin D receptor.^19^

After accounting for age in our study, the difference in 25(OH)D_3_ concentration alone between CM and N groups was not statistically significant. However, mean summation vitD was different between CM and N cat groups, both before and after accounting for age. If summation vitD is a better index of vitamin D status in cats, rather than 25(OH)D_3_ alone, our findings are consistent with studies in people and dogs, which identify lower vitamin D status associated with serious CV disease.^1^, ^2^, ^3^, ^4^, ^5^

We found survival time to be associated with both summation vitD and 25(OH)D_3_, even after accounting for age. However, we did not observe actual survival days beyond the study's end date, meaning this variable is right‐censored. Admittedly, such a limitation could cause bias in model estimation, which is another reason for removing it in the final regression models for 25(OH)D_3_ and summation vitD. Vitamin D status, assessed by 25(OH)D concentration, also was an independent predictor of mortality in a study of 99 sick cats (with various disease conditions) when the analysis used 25(OH)D as a categorical value, although not as a continuous variable.^20^ In that study, cats with 25(OH)D concentrations in the lowest tertile, <73.6 nM/L (ie, <29.4 ng/mL), had higher mortality compared to cats with higher 25(OH)D concentrations, with an odds ratio of 8.27 for death within 30 days. In dogs with DMVD, a significant association between 25(OH)D concentration and time to clinical CHF signs or sudden death was reported.^4^ Lower serum 25(OH)D concentrations also were identified in dogs with chronic enteropathy with death related to their disease.^21^ Multiple studies in people have shown an association between vitamin D deficiency and increased incidence of all‐cause, as well as CV, mortality.^1^, ^2^, ^3^

Declining renal function is known to impair conversion of 25(OH)D_3_ to the active 1,25 (OH_2_)D_3_ (calcitriol) in people.^15^ This effect is exacerbated when the supply of substrate (ie, 25(OH)D) is decreased. Nevertheless, data analysis in our cats showed that azotemia status was related to age and not to vitamin D status, whether assessed by 25(OH)D_3_ alone or by summation vitD.

Vitamin D exerts its effects mainly by activating vitamin D receptors, which function as a transcription factor that regulates various downstream signaling pathways and controls target gene transcription and expression. Vitamin D receptors are expressed in many tissues, including the heart.^1^, ^6^, ^22^ Experimental studies in mice, rats, and swine have shown vitamin D to have several cardioprotective effects, including decreases in renin‐angiotensin activation, myocardial hypertrophy, endothelial dysfunction, and pro‐inflammatory cytokines.^3^, ^22^, ^23^, ^24^ Cardiac contractility and intracellular calcium dynamics also are affected by cardiac vitamin D receptor activation.^24^ Systemically, adequate vitamin D availability also helps decrease inflammation, improve immunity, and could have antioxidant properties.^6^, ^14^, ^17^, ^25^ Adequate vitamin D concentrations have been associated with decreased occurrence of diabetes, CV disease, and certain cancers, among other conditions in people. Vitamin D deficiency is thought to contribute to sustained renin‐angiotensin‐aldosterone axis activation.^2^ Whether vitamin D directly influences mortality or if other systemic effects of low vitamin D status ultimately influence survival is unclear. Likewise, changes in vitamin D status possibly may be secondary to various diseases, rather than a primary cause of morbidity and mortality.

In people, serum 25(OH)D concentrations ≥30 ng/mL are considered sufficient (although possibly not optimal); 25(OH)D concentrations of 20 to 29 ng/mL are considered insufficient, and <20 ng/mL is considered deficient. Concentrations of 25(OH)D that correspond to sufficient, insufficient (suboptimal), or deficient in cats are not well defined. Previous studies in cats have found a wide range of serum 25(OH)D concentrations in normal cats, with reported median concentrations between 44.7 and 49 ng/mL.^6^ The median 25(OH)D_3_ concentration in our N group was slightly lower, at close to 43 ng/mL. Measurement methodology can affect results. Additionally, the contribution of 3‐epi, if any, to determinations of vitamin D sufficiency in cats is unclear.

Most studies of vitamin D status and heart failure in humans, and the 2 reports in dogs, involve conditions likely to be associated with decreased LV systolic function. However, cats with acquired cardiac disease are more likely to have predominantly diastolic dysfunction. Vitamin D is thought to have a protective effect in LV diastolic dysfunction, by inhibiting myocardial fibrosis and enhancing relaxation by modulation of myocardial calcium mechanics.^26^ Conversely, an experimental study in swine identified increased cardiomyocyte hypertrophy, as well as an increase in inflammatory markers in animals with vitamin D deficiency.^22^ In cats with CM, the severity of LAE has been viewed as a clinical indicator of LV diastolic dysfunction. Our study showed a significant negative relationship between summation vitD and LAE severity, even after accounting for age. Few reports of vitamin D status are available in people with predominantly diastolic dysfunction. One study of middle‐aged to older people with diastolic dysfunction, but not specifically HCM, found that those with lower 25(OH)D concentrations had increased hospitalization rates, but only a tendency (*P* = .05) for increased 5‐year mortality.^3^ Patients in the lowest tertile of 25(OH)D concentrations were older, more often symptomatic, and had more severe LAE (calculated as LA volume index) and higher NT‐pro‐brain natriuretic peptide concentrations. Increased LA volume index, among other measures, was independently associated with decreased 25(OH)D. Associations between a higher probability of low 25(OH)D and measures of LV diastolic dysfunction, heart failure, and atrial fibrillation were found, even after adjusting for age.^3^ Others have found a trend toward increased prevalence of diastolic dysfunction in patients with vitamin D deficiency.^27^ However, subsequent evaluation of that same patient cohort (asymptomatic diastolic dysfunction or heart failure with preserved ejection fraction) showed no strong association between 25(OH)D and myocardial structure and function.^28^ We found no significant relationship between LV hypertrophy and vitamin D status in our cats. However, as an indicator of myocardial hypertrophy, our combined LV wall and septal thickness measure was much less sensitive than histologic cardiomyocyte examination.

Diagnosis category provided interesting results in our analysis of summation VitD. The category other CM (consisting of cats with RCM, DCM, and unspecified CM) showed lower mean summation vitD compared to N cats, before age (as a continuous variable) was included in the model. Although significance decreased with age included, application of the age group filter showed that summation vitD was lower in older (group 3) cats with the other CM classification. This category included CM types generally considered to have more advanced pathophysiology, involving either diastolic or systolic dysfunction, or a combination. From this standpoint, the finding is similar to reports of lower vitamin D status in people (and dogs) with more advanced disease.

Calculation of FS (or ejection fraction) often is a clinical proxy for LV systolic function. Our data showed FS was significantly related to 25(OH)D_3_ and summation vitD concentrations, even after accounting for age. Decreased systolic function is likely in cats with RCM and end‐stage HCM, as well as in those with DCM. In people, worse vitamin D status is more likely in those with advanced heart failure.

Most of our CM cats had experienced an episode of CHF at some point in time. The majority of these had evidence for congestion (either pulmonary edema or pleural effusion) at the time of study entry. Based on studies in people and dogs with heart failure, we expected to find lower vitamin D status in these cats. We found no association between heart failure status and 25(OH)D_3_. A negative relationship between summation vitD and heart failure severity was detected before accounting for age but, after including age in the model, the significance became only borderline. Presumably, older cats are more likely to develop CHF, as well as to have lower vitamin D status, and thus age as a confounder could be responsible for the change in significance.

Our study had several limitations. One limitation is that we were not able to control dietary intake of vitamin D in our cats. An entry criterion was that the primary diet be a commercially available diet, for which vitamin D content should be available from the manufacturers. However, it was not possible to quantitate the exact amounts of different foods (and flavors) eaten daily by each cat and not all manufacturers were able to supply vitamin D information. Therefore, the estimation of dietary vitamin D intake was incomplete. Besides variability in the types of food fed, some cats had free‐choice access, often with other cats in the household, so their food intake could not be accurately quantified. Furthermore, we could not quantitate vitamin D intake from table food, cat treats, or other foods that might have been given to (or found by) the cats, although these comprised only a minor proportion of the diet in this population. In addition, because we sampled these cats at only 1 time point, we have no way of knowing whether changes in vitamin D concentration over time might occur relative to disease progression or dietary change in CM cats. Likewise, we do not know if PO vitamin D supplementation would affect disease progression or survival.

Given the substantial circulating concentrations of 3‐epi in cats, we suspect that summation vitD could be a better index of vitamin D status in this species, compared to 25(OH)D_3_ alone. However, further study is needed to determine if this truly is the case. The most biologically active form of vitamin D is calcitriol (1, 25 (OH_2_)D_3_), which we did not measure. Likewise, we did not attempt to measure the epimer of calcitriol, which experimental studies have indicated has some biological effect. Additional research will be important to clarify the effects and relationships among these vitamin D metabolites in cats.

In conclusion, we report on 25(OH)D_3_ and its C‐3 epimer (3‐epi) concentrations in cats with CM and in clinically healthy cats. Notable concentrations of 3‐epi are present in this species, in contrast to the situation in people and dogs. Therefore, 3‐epi could contribute importantly to the clinical assessment of total vitamin D status in cats. In addition, we found that age had a significant negative effect on vitamin D status, whether assessed by 25(OH)D_3_ alone or by the sum of 25(OH)D_3_ and 3‐epi concentrations (ie, summation vitD). After accounting for age, summation vitD was lower in CM compared to N cats. Vitamin D status also was positively related to survival time and FS, but negatively related to LAE severity.

## CONFLICT OF INTEREST DECLARATION

Andrew J. Makowskic is employed by Heartl and Assays. No other authors have a conflict of interest.

## OFF‐LABEL ANTIMICROBIAL DECLARATION

Authors declare no off‐label use of antimicrobials.

## INSTITUTIONAL ANIMAL CARE AND USE COMMITTEE (IACUC) OR OTHER APPROVAL DECLARATION

The study was approved by the animal care and use committees at Tufts University and Iowa State Univeristy.

## HUMAN ETHICS APPROVAL DECLARATION

Authors declare human ethics approval was not needed for this study.

## Supporting information


**Data S1** Supporting Information
