# Supplementary material for: Vitamin D status in cats with cardiomyopathy
Source: J Vet Intern Med. 2020 Jun 17;34(4):1389–98. doi: 10.1111/jvim.15833 (PMC7379033; doi:10.1111/jvim.15833)
Supplement: Supplementary file 1 — Data S1 Supporting Information [file JVIM-34-1389-s001.pdf]

Vitamin D Status in Cats – Owner Questionnaire

ISU Med Rec # ________________

Owner Name (Last, First) ___________________________________ Phone # to reach you at today: __________

Date _______________ Cat's Name _____________________

How active is your pet? __ very active __ moderately active __ not very active

How would you describe your pet's weight? __ overweight __ ideal weight __underweight

Where does your pet spend most of the time? __ indoors __ outdoors __ indoors & outdoors

Does your cat eat a commercial cat food for at least 80% of his/her total diet? Yes ___ No ____

Diet information (please fill in the information requested as completely as possible):

Food brand & specific product	Flavor	Dry (D) or Canned (C)?	Amount* fed/meal	Number of meals/day	Fed since (month/year)

*If you feed by volume, what size measuring device do you use? ____
If you feed canned food, what size can (in ounces)?

Do you give any dietary supplements? __ No __ Yes, please list brands & amounts _______________________

Has your cat been diagnosed with cardiomyopathy or other cardiac disease? Yes ___ No ____

If yes, please describe any symptoms. (If on medications, please list below or on back)

Has your cat been diagnosed with high blood pressure? Yes ___ No ____

If yes, please describe any symptoms. (If on medications, please list below or on back)

Does your cat have any other medical problem(s)? Yes ___ (please describe below or on back) No ____

Current Medications:

Drug Name	Size/Strength	Dose/Frequency
1.		
2.		
3.		
4.		

May we shave a small amount of hair from your pet if necessary for echocardiography, blood pressure, venipuncture, etc.?

Yes _____ No _____

Do you give permission for mild sedation to be given if necessary for echocardiography? Yes _____ No _____
